# Different Effects of SSRIs, Bupropion, and Trazodone on Mitochondrial Functions and Monoamine Oxidase Isoform Activity

**DOI:** 10.3390/antiox12061208

**Published:** 2023-06-02

**Authors:** Matej Ľupták, Zdeněk Fišar, Jana Hroudová

**Affiliations:** 1 Institute of Pharmacology, First Faculty of Medicine, Charles University and General University Hospital in Prague, Albertov 4, 128 00 Prague, Czech Republic; 2 Department of Psychiatry, First Faculty of Medicine, Charles University and General University Hospital in Prague, Ke Karlovu 11, 120 00 Prague, Czech Republic; zfisar@lf1.cuni.cz

**Keywords:** oxidative phosphorylation, mitochondrial respiration, reactive oxygen species, ATP, monoamine oxidase, antidepressants

## Abstract

Mitochondrial dysfunction is involved in the pathophysiology of psychiatric and neurodegenerative disorders and can be used as a modulator and/or predictor of treatment responsiveness. Understanding the mitochondrial effects of antidepressants is important to connect mitochondria with their therapeutic and/or adverse effects. Pig brain-isolated mitochondria were used to evaluate antidepressant-induced changes in the activity of electron transport chain (ETC) complexes, monoamine oxidase (MAO), mitochondrial respiratory rate, and ATP. Bupropion, escitalopram, fluvoxamine, sertraline, paroxetine, and trazodone were tested. All tested antidepressants showed significant inhibition of complex I and IV activities at high concentrations (50 and 100 µmol/L); complex II + III activity was reduced by all antidepressants except bupropion. Complex I-linked respiration was reduced by escitalopram >> trazodone >> sertraline. Complex II-linked respiration was reduced only by bupropion. Significant positive correlations were confirmed between complex I-linked respiration and the activities of individual ETC complexes. MAO activity was inhibited by all tested antidepressants, with SSRIs causing a greater effect than trazodone and bupropion. The results indicate a probable association between the adverse effects of high doses of antidepressants and drug-induced changes in the activity of ETC complexes and the respiratory rate of mitochondria. In contrast, MAO inhibition could be linked to the antidepressant, procognitive, and neuroprotective effects of the tested antidepressants.

## 1. Introduction

Despite progress in neuroscience research over the past decades, major depressive disorder (MDD) pathophysiology has not been fully clarified. Understanding the effects antidepressants and other psychotropic drugs at the molecular level could provide new knowledge on pathophysiology and aid in the search for antidepressants with new mechanisms of action. Historically, depression has been viewed as a multifactorial disorder linked to different biochemical and physiological disturbances in neurotransmitter systems, neuroendocrine and immune functions, circadian rhythms, synaptic and structural neuroplasticity, and neuronal adaptation. Recent evidence includes the association of disturbances in neuroplasticity and brain function with mitochondrial dysfunction and inflammation in the pathogenesis of MDD and the influence of these processes by drugs [1,2].

**Figure 1:**
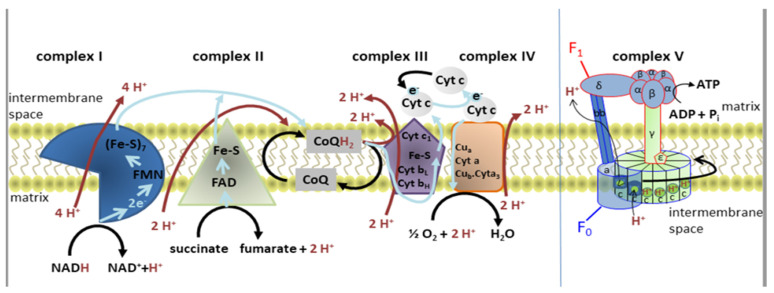
Diagram of the electron transport chain and the structure of ATP synthase. Reduced nicotinamide adenine dinucleotide (NADH) donates electrons (blue arrows) that pass through complex I via flavin mononucleotide (FMN) and iron-sulfur (Fe-S) clusters. Together with two protons, they bind to oxidized coenzyme Q (CoQ) to form reduced coenzyme Q (CoQH_2_). This electron flow allows four H^+^ (red arrows) to be transported into the intermembrane space. Complex II is the electron side entrance, as electrons from succinate pass through oxidized flavin adenine dinucleotide (FAD^+^) and Fe-S before forming CoQH_2_. Electrons then flow from CoQH_2_ to complex III, and through cytochrome c (Cyt c) to complex IV. In total, ten H^+^ are transported into the intermembrane space for each NADH or six H^+^ for each FADH_2._ The stator (blue) and the rotor (green) form complex V. The F_O_ domain of ATP synthase consists of three a subunits, three b subunits, and ten c subunits forming the c-ring. The a subunit contains the H^+^ ion half-channel, which is responsible for mediating proton movement across the membrane. The α and β subunits of F_1_ form a hexamer on the top of the γ subunit, which is inserted into the c-ring. Adapted from Ľupták et al. [3].

Mitochondria are the main energy suppliers for all cellular processes in the organism. In addition to being sources of energy, mitochondria are crucial organelles in balancing calcium levels, reactive oxygen species (ROS) production, and apoptosis regulation, key processes in neurodevelopment and neuroplasticity. The mitochondrial electron transport chain and the structure of ATP synthase are shown in Figure 1 [3]. There is a large body of evidence implying that mitochondrial dysfunction is one of the key players responsible for the development of MDD and other psychiatric and neurodegenerative disorders (including schizophrenia, Parkinson’s disease, and Alzheimer’s disease). Disruption of mitochondrial functions leads to depletion in ATP production, pushing the brain and other high-energy-demanding tissues toward energetic imbalance, endangering the maintenance of cellular tissue and body homeostasis. Mitochondrial dysfunction can have far-reaching consequences, e.g., increased oxidative stress, inaccurate neuronal signaling, and neuroinflammation, which all inevitably lead to aggravated neuronal adaptability and inefficient maintenance of body homeostasis [1,2,4,5,6]. Preclinical studies observing rat and mouse models of depression showed decreased ATP production [7], altered brain respiratory rate and membrane potentials, and damaged brain mitochondrial ultrastructure [8]. Rappeneau et al. more closely mapped the correlates of mitochondrial dysfunction in MDD in both clinical and preclinical studies [9]. Multiple studies have shown the presence of mitochondrial dysfunction in MDD patients, observed as abnormal energy metabolism in several brain regions [10,11,12,13,14,15], decreased mitochondrial respiration and altered activity of mitochondrial electron transport chain (ETC) complexes and other mitochondrial functions [16,17], oxidative imbalance [14], altered mtDNA copy number, and mtDNA mutations [18,19].

Approximately 50% of treated MDD patients achieve remission. It was previously proposed by Emmerzaal et al. that mitochondrial dysfunction could be one of the modulators of treatment response. Evaluating patients’ mitochondrial functions and the bioenergetic profile of psychiatric/neurodegenerative disorders might help in understanding the unique bioenergetics of individuals. This would also help with personalized pharmacotherapy of psychiatric diseases when the most appropriate medication (especially for pharmacoresistant patients) could be chosen. Identifying potential modulators of treatment response (e.g., mitochondrial dysfunction) could be a reasonable step toward personalized pharmacological approaches for psychiatric and neurodegenerative diseases [20]. The impact of antidepressants on mitochondrial functions has shown both positive and negative effects. Fernström et al. showed that mitochondrial markers might help distinguish selective serotonin reuptake inhibitor (SSRI) responders and nonresponders (patients were taking sertraline, fluoxetine, citalopram, and escitalopram). SSRI responders had significantly higher citrate synthase (CS) baseline levels and complex I activity, which decreased with treatment (complex I activity increased with treatment in nonresponders). Treatment-associated changes, evaluated with the Hamilton Depression Rating Scale, correlated with changes in complex I activity. Complex II activity increased with SSRI administration [21].

**Figure 2:**
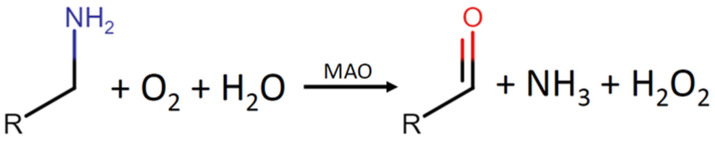
Enzymatic reactions catalyzed by monoamine oxidase (adapted from Fišar et al.) [23]. Monoamines (e.g., dopamine, serotonin, or norepinephrine) are metabolized by oxidative deamination (along with oxygen and water) catalyzed by monoamine oxidase (MAO) to form aldehyde, ammonia, and hydrogen peroxide. Subsequently, aldehyde dehydrogenase oxidizes aldehydes into carboxylic acids, and hydrogen peroxide can be further converted to hydroxyl radical.

The mitochondrial sources of ROS include not only complex I and complex III of the electron transport chain (ETC) but also the enzyme monoamine oxidase (MAO), which is located on the outer membrane of mitochondria and catalyzes the oxidative deamination of biogenic amines, including monoamine neurotransmitters [22]. The scheme of enzymatic reactions catalyzed by MAOs is shown in Figure 2. MAO is one of the primary sources of ROS in the brain and can participate in increased oxidative stress leading to neurodegeneration, apoptosis and impaired neuroplasticity and neurogenesis, contributing to psychiatric and neurodegenerative diseases [23]. MAO, as a modulator of monoaminergic neurotransmission, could be a target for drugs used in the treatment of neuropsychiatric disorders [23]. The role of MAO in the pathophysiology of MDD is supported by evidence that MAO-A density is increased during the acute phase of MDD [24].

It has been shown that long-term therapy with monoamine oxidase inhibitor (MAOI) leads to neuronal adaptive changes such as membrane receptor density and sensitivity modulation, activity of transporters, transcription factors and intracellular signaling pathways, and increases in neuroplasticity and neurogenesis, neurotrophic factor gene expression and antiapoptotic effects [23,25,26]. MAO also participates on amyloid beta aggregation. MAO inhibition leads to decreased monoamine neurotransmitter metabolism and decreased ROS production, both of which are mechanisms of action of antidepressants and drugs for the treatment of Alzheimer’s or Parkinson’s disease. The neuroprotective properties of some MAOIs are probably linked to their antiapoptotic effects and modulation of gene expression [6,23,27,28]. MAO-B inhibitors (selegiline, rasagiline) are preferentially used in pharmacotherapy for Parkinson’s disease; they balance oxidative stress and induce the production of antiapoptotic Bcl-2 and neurotrophic factors. Irreversible MAO-A inhibitors (phenelzine, clorgyline, pargyline) were some of the first antidepressants. Severe adverse effects and frequent interactions led to their withdrawal from the market and their subsequent replacement by safer SSRIs, the first-choice antidepressants [23,27,28]. However, it has been estimated that 15–20% of MDD patients require nonselective MAOIs (phenelzine, tranylcypromine) as a part of antidepressant therapy to achieve an optimal response [29].

SSRIs selectively inhibit serotonin reuptake, and their antidepressant effect is mediated via modulation of serotonergic transmission. In addition to MDD, SSRIs are also indicated for anxiety disorders, posttraumatic stress disorder, obsessive compulsive disorder, and bulimia. These antidepressants share similar adverse effects, including gastrointestinal irritation, anxiety, sexual dysfunctions, impaired cognition, increased risk of suicidal thoughts and actions, and risk of serotonin syndrome [30]. Escitalopram (ESC) is the therapeutically active S-enantiomer of citalopram and the most selective SSRI; it displays rapid onset of antidepressant action [31]. Fluvoxamine (FLUV) is structurally different from other SSRIs and has higher selective inhibitory properties toward presynaptic uptake of serotonin, making it especially beneficial for obsessive-compulsive and bulimic patients. According to binding studies, paroxetine (PAR) is the most potent inhibitor of serotonin reuptake, with low norepinephrine uptake and low affinity toward cholinergic receptors. It is suitable for use in elderly patients, and in addition to its psychiatric indications, it is also used for the treatment of chronic headache and premenstrual dysphoria disorder. Sertraline (SER) is a highly selective SSRI with low potency toward norepinephrine and dopamine transporters and low affinity to cholinergic, histaminergic, and noradrenergic receptors. It is also used for premenstrual dysphoric syndrome and in premature ejaculation therapy. SER possess the highest risk of suicidality among this group of medications [30].

Bupropion (BUP) and trazodone (TRA) are both non-SSRI antidepressants. BUP acts as a norepinephrine and dopamine reuptake inhibitor and nicotinic acetylcholine receptor antagonist. It is used as an antidepressant and for smoking cessation treatment and for obesity therapy [32]. TRA acts as a serotonin antagonist and reuptake inhibitor; it antagonizes serotonin type 2 and alpha-adrenoreceptors and inhibits serotonin reuptake. It is used for the treatment of MDD, insomnia, anxiety disorders, and sexual dysfunction [33].

The aim of this study was to investigate the effects of selected SSRIs (ESC, FLUV, PAR, and SER), BUP and TRA on mitochondrial functions and the activities of MAO-A and MAO-B. Mitochondrial dysfunction and MAO are connected to the pathophysiology of neurodegenerative and psychiatric disorders. Understanding the effects of the chosen antidepressants on mitochondrial complexes and enzymes could provide deeper insight into their overall influence on mitochondrial function and cell energy metabolism.

## 2. Materials and Methods

All materials and methods have been described in our previous article [34]; only a summary follows.

### 2.1. Media and Chemicals

Sucrose 0.32 mol/L and HEPES 4 mmol/L (pH 7.4) were used as the mitochondrial isolation medium. The respiratory medium (MiR05 without BSA) contained sucrose 110 mmol/L, K^+^-lactobionate 60 mmol/L, taurine 20 mmol/L, MgCl_2_·6H_2_O 3 mmol/L, KH_2_PO_4_ 10 mmol/L, EGTA 0.5 mmol/L, and HEPES 20 mmol/L (pH 7.1). The Krebs–Henseleit (KH) buffer consisted of NaCl 118 mmol/L, KCl 4.7 mmol/L, KH_2_PO_4_ 1.2 mmol/L, NaHCO_3_ 25 mmol/L, and glucose 11.1 mmol/L. 2-phenylethylamine [ethyl-1-^14^C] hydrochloride ([^14^C]PEA) and 5-Hydroxytryptamine [^3^H] trifluoroacetate ([^3^H]serotonin) were bought from American Radiolabeled Chemicals, (St. Louis, MO, USA), and other substances and chemicals were bought from Sigma-Aldrich Co. (St. Louis, MO, USA).

### 2.2. Isolation of Pig Brain Mitochondria

The mitochondrial fraction was isolated and purified from the pig brain cortex [35]. For high-resolution respirometry and ATP assays, freshly prepared mitochondria were kept on ice. Part of the mitochondria was frozen, stored at −70 °C, and used to determinate mitochondrial enzymes activities (ETC complexes, CS, malate dehydrogenase, and MAO).

### 2.3. Mitochondrial Enzymes Activities

Ultrasonicated mitochondria were incubated with the tested drugs for 30 min at 30 °C, with a corresponding drug-free control for every measurement. The activities of mitochondrial enzymes were determined as the absorbance measured with a GENESYS 180 UV-Vis spectrophotometer (Thermo Fisher Scientific, Waltham, MA, USA).

#### 2.3.1. Activity of Citrate Synthase

Tris, Triton 5,5′-dithiobis-(2-nitrobenzoic) acid, and acetyl coenzyme A were mixed as the reaction mixture. The reaction was started by adding oxaloacetate and the activity of CS was detected as the change in absorbance of 5,5′-dithiobis-(2-nitrobenzoic) acid (measured at 412 nm for 3 min). The final concentration of all tested drugs was 100 µmol/L, and the final protein concentration was 20 µg/mL [36].

#### 2.3.2. Activity of Malate Dehydrogenase

The conversion of oxaloacetate to malate was used to measure malate dehydrogenase (MDH) activity. The reaction was started by adding oxaloacetate and nicotinamide adenine dinucleotide (NADH) (measured at 340 nm for 3 min). The final concentration of all tested drugs was 100 µmol/L, and the final protein concentration was 20 µg/mL [37].

#### 2.3.3. Activity of Complex I (NADH Dehydrogenase)

KH_2_PO_4_, MgCl_2_, and KCN were mixed as the reaction mixture. The reaction was started by adding decylubiquinone and NADH. Oxidation of NADH was measured for 5 min at 340 nm. The final concentrations of all tested drugs were 2.5, 5, 10, 50, and 100 µmol/L, and the final protein concentration was 150 µg/mL [38].

#### 2.3.4. Activity of Complex II+III (Succinate Cytochrome *c* Oxidoreductase)

The activity of complex II+III was measured as the reduction in cytochrome *c* (measured at 550 nm for 3 min). The reaction was started by adding cytochrome *c*. KH_2_PO_4_, EDTA, KCN, and rotenone were mixed as the reaction mixture. The final concentrations of all tested drugs were 2.5, 5, 10, 50, and 100 µmol/L, and the final protein concentration was 50 µg/mL [39].

#### 2.3.5. Activity of Complex IV (Cytochrome *c* Oxidase)

Reduced cytochrome *c* was added to KH_2_PO_4_ to initiate the reaction. The resulting decrease in absorbance was measured at 550 nm for 3 min. The final concentrations of all tested drugs were 10, 50, and 100 µmol/L, and the final protein concentration was 10 µg/mL [40].

### 2.4. ATP Content and Kinetics

ATP content and kinetics were measured using the ATP Bioluminescence Assay Kit CLS II. Luminescence was measured at 562 nm with FluoroMax-3 (Jobin Yvon, Edison, NJ, USA). An ATP standard curve was constructed using ATP standards in the range of 0 to 600 nmol/L. The final concentrations of all tested drugs were 10, 50, and 100 µmol/L, and the final protein concentration was 50 µg/mL.

#### 2.4.1. Total Complex I- and Complex II-Linked ATP Content

MiR05 buffer was used as the medium and the reaction was started by adding a substrate mix consisting of malate 5 mmol/L and pyruvate 5 mmol/L (for complex I) or succinate 5 mmol/L and rotenone 1 μmol/L (for complex II), ADP 60 μmol/L and MgCl_2_·6H_2_O 0.75 mmol/L. The reaction was stopped by heat and the final complex I- and complex II+III-linked ATP content was determined by luminescence (measured for 1 min) [41].

#### 2.4.2. Complex I- and Complex II-Linked ATP Kinetics

MiR05 buffer was used as the medium, the same substrate mixture as above was added, 230 µL of luciferase reagent was added and ATP kinetics was measured using the luminescence (measured for 4 min) [42].

### 2.5. Mitochondrial Respiration

The high-resolution Oxygraph-2k (Oroboros Instruments Corp, Innsbruck, Austria) was used to measure mitochondrial oxygen consumption rate. Malate 2 mmol/L, pyruvate 5 mmol/L, ADP 1.25 mmol/L, and MgCl_2_ 0.75 mol/L were mixed to form a reaction mixture for complex I-linked respiration; and ADP 1.25 mmol/L, MgCl_2_ 0.75 mol/L, rotenone 1 µmol/L, and succinate 10 mmol/L for complex II-linked respiration. The final protein concentration was 0.05–0.14 mg/mL. Four simultaneous measurements were performed: two oxygraphy chambers were used for a titration up to the final drug concentrations of 0.125–100 µmol/L, and two other oxygraphy chambers were used for a titration with the DMSO as a control [43,44].

### 2.6. Activity of Monoamine Oxidase

KH buffer was used to preincubate mitochondria (final concentration of 800 µg/mL) with the tested drugs (concentration range of 0.1–300 µmol/L). MAO enzymatic activity was measured using radiolabeled substrates ([^3^H]serotonin for MAO-A and [^14^C]PEA for MAO-B). The reaction was terminated with hydrochloric acid, the organic phase was separated, and the radioactivity was determined by liquid scintillation counting (LS 6000IC, Beckman Instruments, Inc., Fullerton, CA, USA) [45,46].

### 2.7. Data Analysis and Statistics

Mitochondrial enzyme activities and ATP kinetics were determined from time-dependent changes in absorbance or fluorescence slope. The ATP content was measured, and the average fluorescence curves over time were calculated. The activity of the control sample was considered to be 100%, and the effect of the drug was expressed as a percentage of the control. An ATP standard curve was constructed.

High-resolution respirometry data were analyzed and real-time oxygen concentrations and flux were displayed using DatLab 7.4 software from Oroboros Instruments (Innsbruck, Austria). Respiration rate (oxygen flux) was quantified as the number of pmol of oxygen consumed per second per mg of a protein.

Four-parametric logistic regression was used to analyze inhibition of respiratory rate and MAO activity to determine half-maximal inhibitory concentration (IC_50_), residual activity, and Hill slope. Prism software from GraphPad (San Diego, CA, USA) was used for this analysis. The IC_50_ indicates the concentration of a drug that is required to reduce the mitochondrial oxygen flux or MAO activity by 50% of the difference between the baseline and the residual value.

Data were analyzed using one-sample *t*-tests in the STATISTICA 12 analysis software (TIBCO Software Inc., Palo Alto, CA, USA). All data presented are expressed as either the mean ± standard deviation (SD) or the mean ± standard error of the mean (SEM). The Pearson correlation coefficient was used to identify statistically significant correlations, which are presented in the correlation matrix. Correlations with total ATP content and kinetics are part of the supplementary material.

## 3. Results

### 3.1. Activity of Mitochondrial Enzymes

**Figure 3:**
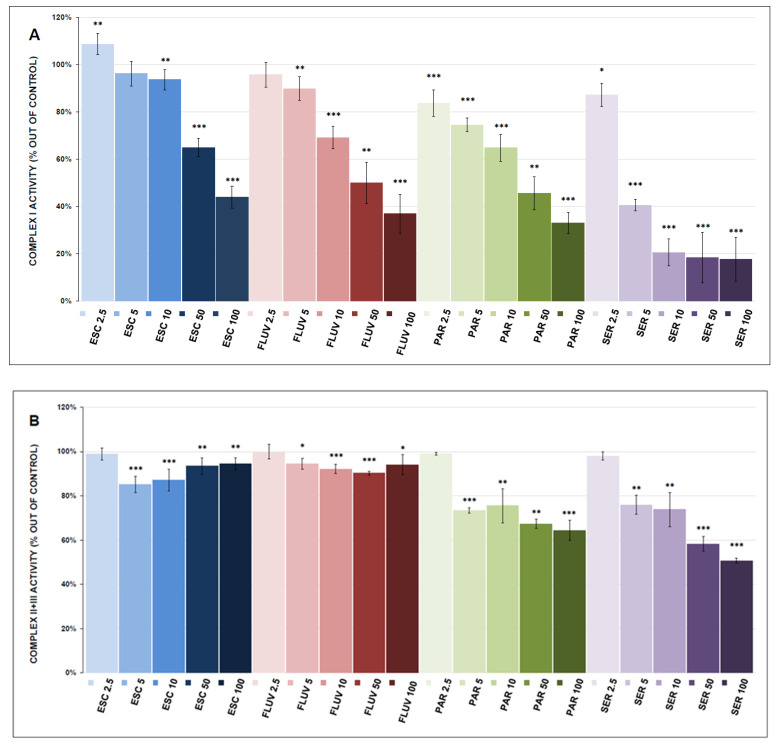

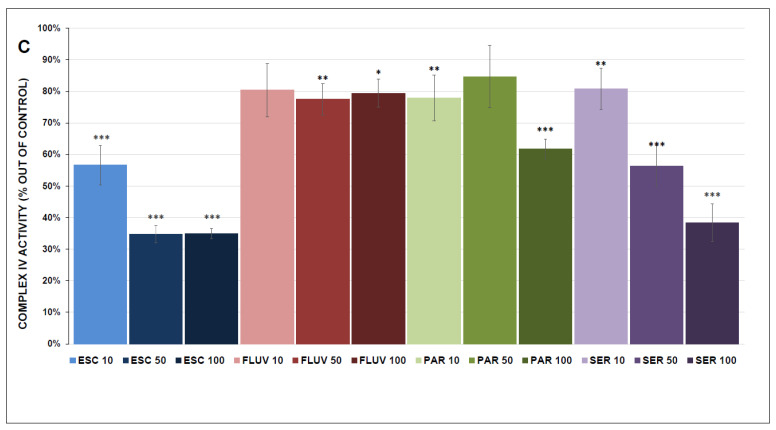
SSRI-induced changes in the activity of electron transport chain (ETC) complexes (complex I, complex II + III and complex IV, (**A**–**C**), respectively). Relative activity is expressed as the percentage difference from the activity of the control sample, with the mean value and standard deviation (SD) calculated from at least three independent measurements. A one-sample *t*-test was performed to assess statistical significance, with the mean control value set at 100% and is expressed as * *p* ˂ 0.05. ** *p* ˂ 0.01. *** *p* ˂ 0.001. Drug concentrations are expressed in µm/L. ESC—escitalopram, FLUV—fluvoxamine, PAR—paroxetine, SER—sertraline.

**Figure 4:**
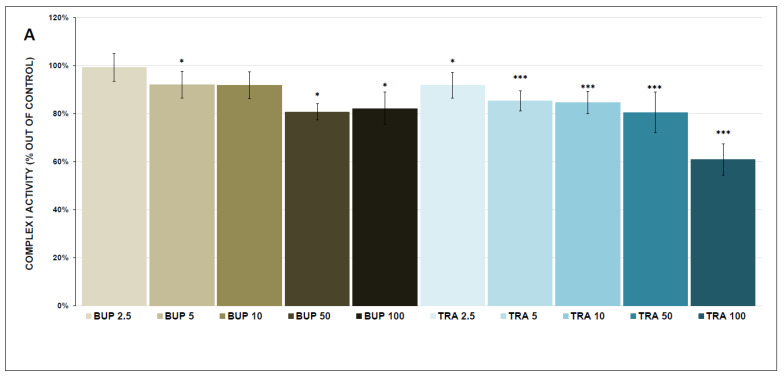

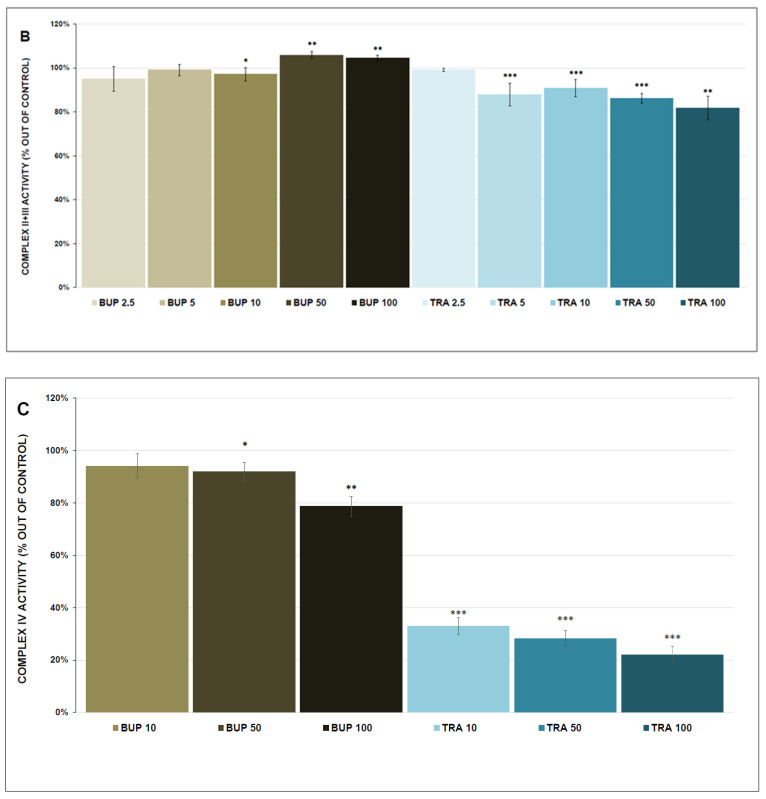
Bupropion- and trazodone-induced changes in the activity of electron transport chain (ETC) complexes (complex I, complex II + III and complex IV, (**A**–**C**), respectively). Relative activity is expressed as the percentage difference from the activity of the control sample, with the mean value and standard deviation (SD) calculated from at least three independent measurements. A one-sample *t*-test was performed to assess statistical significance, with the mean control value set at 100% and is expressed as * *p* ˂ 0.05. ** *p* ˂ 0.01. *** *p* ˂ 0.001. Drug concentrations are expressed in µm/L. BUP—bupropion, TRA—trazodone.

The results of the assessments of mitochondrial respiratory complex activity are shown in Figure 3A–C and Figure 4A–C. All tested doses of antidepressants (except ESC 2.5 µmol/L) inhibited complex I activity (significantly at most concentrations). SSRI antidepressants showed more potent inhibition. The antidepressants are listed from the most potent to the least potent: SER (17.5 ± 9.3% at 100 μmol/L, *p* < 0.001), PAR (33.0 ± 4.4% at 100 μmol/L, *p* < 0.001), FLUV (36.8 ± 8.2% at 100 μmol/L, *p* < 0.001), ESC (43.9 ± 4.7% at 100 μmol/L, *p* < 0.001), TRA (60.9 ± 6.6% at 100 μmol/L, *p* < 0.001), and BUP (82.1 ± 6.8% at 100 μmol/L, *p* = 0.045).

Complex II+III activity was inhibited by all tested antidepressants except BUP, which showed weak stimulatory activity (104.5 ± 1.2% at 100 μmol/L, *p* = 0.013). The most potent inhibitor was SER (50.7 ± 1.2% at 100 μmol/L *p* < 0.001), followed by PAR (64.4 ± 4.5% at 100 μmol/L, *p* < 0.001), TRA (81.7 ± 5.5% at 100 μmol/L, *p* = 0.002), FLUV (94.1 ± 4.6% at 100 μmol/L, *p* = 0.044), and ESC (94.4 ± 2.7% at 100 μmol/L, *p* = 0.002).

Complex IV activity was inhibited by all tested antidepressants: TRA (22.0 ± 3.3% at 100 μmol/L, *p* < 0.001), ESC (34.9 ± 1.7% at 100 μmol/L, *p* < 0.001), SER (38.4 ± 1.7% at 100 μmol/L, *p* < 0.001), PAR (61.1 ± 3.1% at 100 μmol/L, *p* < 0.001), BUP (78.6 ± 3.8% at 100 μmol/L, *p* = 0.001), and FLUV (79.4 ± 4.4% at 100 μmol/L, *p* = 0.015).

None of the tested antidepressants affected CS and MDH activities (the results are shown in Table S1 of the Supplementary Material).

### 3.2. Mitochondrial Respiration

**Figure 5:**
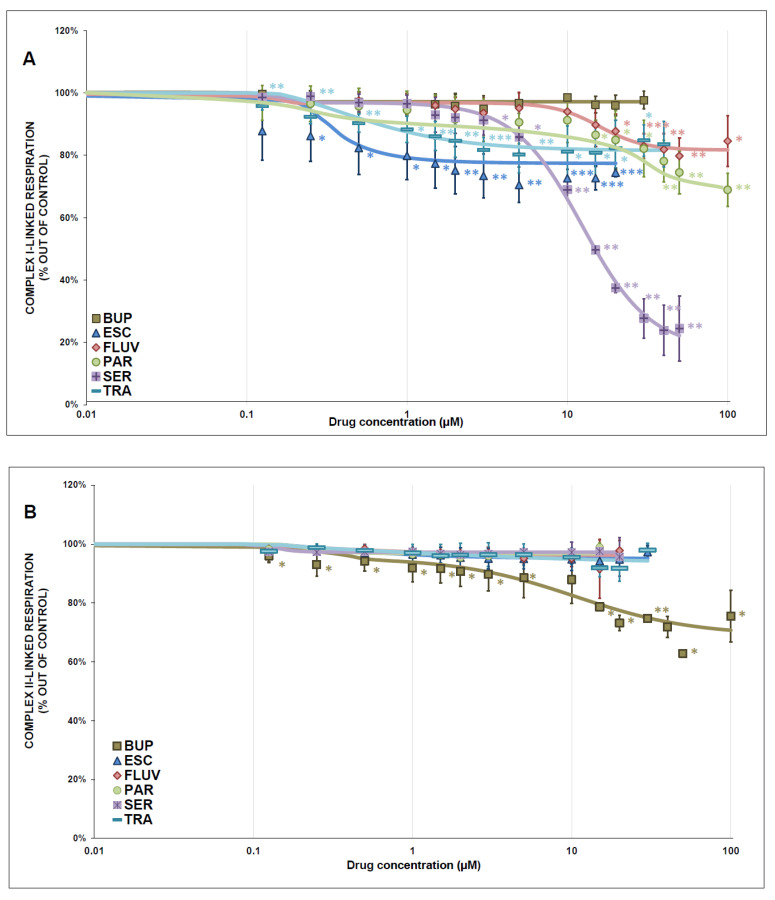
Effect of antidepressants on respiration in isolated mitochondria (complex I-linked respiration, complex II-linked respiration, (**A**,**B**), respectively). The dose-response curves are represented by the oxygen consumption rate plotted against drug concentration. The respiratory rate of the sample titrated with the antidepressants is relative to the control sample titrated with the DMSO (solvent). Four different measurements were used to calculate the plot points. Statistical significance was tested using a one-sample *t*-test with a mean control value of 100%, indicated as * *p* ˂ 0.05. ** *p* ˂ 0.01. *** *p* ˂ 0.001. Table 1 shows the half-maximal inhibitory concentration (IC_50_), Hill slope, and the residual activity calculated using a four-parameter logistic function. BUP—bupropion, ESC—escitalopram, FLUV—fluvoxamine, PAR—paroxetine, SER—sertraline, TRA—trazodone.

The drug-induced inhibition of complex I- and complex II-linked respiration is shown in Figure 5A,B, and the evaluated parameters (IC_50_, Hill slope and residual activity) are summarized in Table 1 (for partial inhibitors only). All tested substances acted as partial inhibitors of complex I-linked respiration. SER was the most potent complex I-linked respiration inhibitor, with a respiration rate of 24.5 ± 10.4% (mean ± SD) at 50 μmol/L (*p* < 0.001), an IC_50_ of 12.4 ± 0.8 μmol/L (mean ± SEM), and a residual activity of 17.9% ± 0.03 (mean ± SEM). As mentioned previously, drug titration was stopped at the point of sudden increase in the mitochondrial respiratory rate, which occurred at high drug concentrations [34].

BUP was the only tested antidepressant that acted as a weak partial inhibitor of complex II-linked mitochondrial respiration. Other antidepressants showed very weak or no inhibitory activity.

### 3.3. ATP Content and Kinetics

The complex I-linked ATP content was significantly decreased by 50 μmol/L TRA (87.8 ± 0.4%, *p* = 0.013) and increased by SER (118.0 ± 6.0% at 50 μmol/L, *p* = 0.009) and 10 μmol/L TRA (111.3 ± 3.0%, *p* = 0.022). The complex I-linked ATP kinetics were significantly inhibited by ESC (81.6 ± 5.1% at 50 μmol/L, *p* = 0.006), FLUV (80.7 ± 10.7% at 50 μmol/L, *p* = 0.037), and PAR (80.4 ± 5.1% at 10 μmol/L, *p* = 0.022).

The complex II-linked ATP content was significantly decreased by PAR (88.6 ± 4.0% at 100 μmol/L, *p* = 0.040). The complex II-linked ATP kinetics were significantly stimulated by BUP (105.7 ± 0.4% at 50 μmol/L, *p* = 0.032) and inhibited by ESC (81.5 ± 4.5% at 50 μmol/L, *p* = 0.019), FLUV (76.6 ± 5.4% at 50 μmol/L, *p* = 0.017), PAR (82.1 ± 0.1% at 100 μmol/L, *p* = 0.004), SER (82.9 ± 2.0% at 10 μmol/L, *p* = 0.005), and TRA (87.6 ± 3.0% at 10 μmol/L, *p* = 0.019).

**Figure 6:**
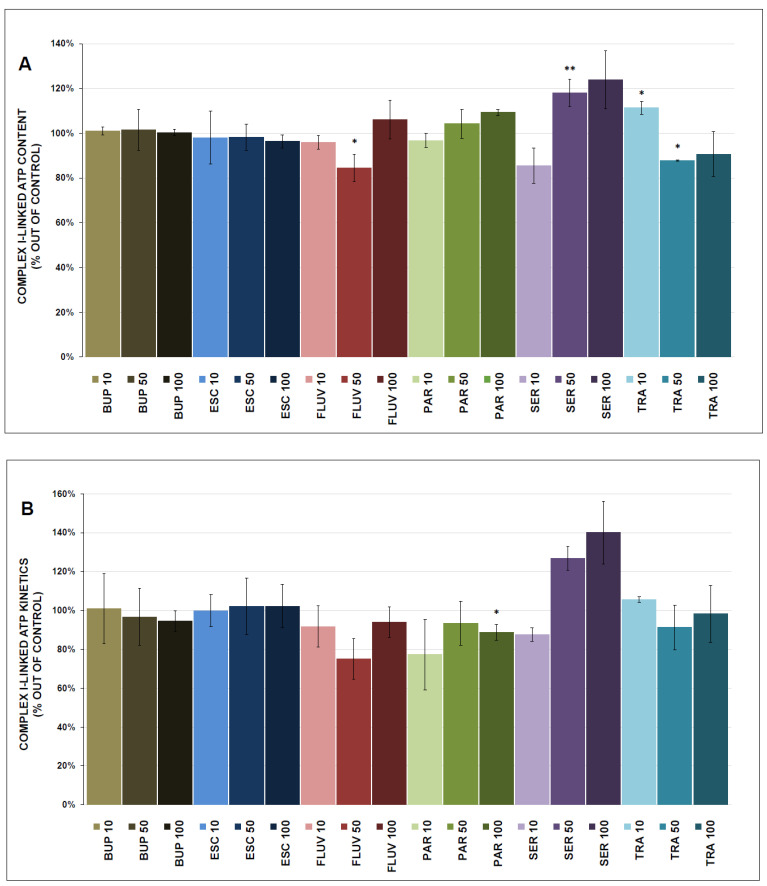
Antidepressant-induced changes in complex I-linked ATP content and kinetics (ATP content, ATP kinetics, (**A**,**B**), respectively). Relative activity is expressed as the percentage difference from the activity of the control sample (100% corresponded to the production of (**A**) 160 nmol of ATP per 1 mg of protein and (**B**) 282 nmol of ATP per 1 mg of protein per 1 min), with the mean value and standard deviation (SD) calculated from at least six independent measurements. A one-sample *t*-test was performed to assess statistical significance, with the mean control value set at 100% and is expressed as * *p* ˂ 0.05. ** *p* ˂ 0.01. Drug concentrations are expressed in µm/L. BUP—bupropion, ESC—escitalopram, FLUV—fluvoxamine, PAR—paroxetine, SER—sertraline, TRA—trazodone.

**Figure 7:**
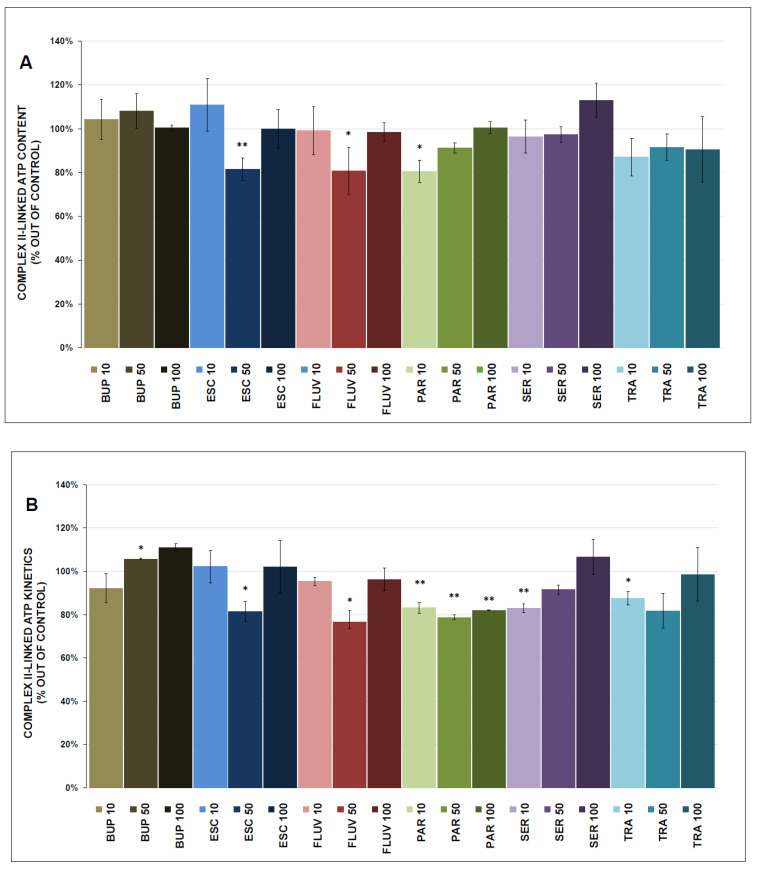
Antidepressant-induced changes in complex II-linked ATP content and kinetics (ATP content, ATP kinetics, (**A**,**B**), respectively). Relative activity is expressed as the percentage difference from the activity of the control sample (100% corresponded to the production of (**A**) 291 nmol of ATP per 1 mg of protein and (**B**) 1289 nmol of ATP per 1 mg of protein per 1 min), with the mean value and standard deviation (SD) calculated from at least six independent measurements. A one-sample *t*-test was performed to assess statistical significance, with the mean control value set at 100% and is expressed as * *p* ˂ 0.05. ** *p* ˂ 0.01. Drug concentrations are expressed in µm/L. BUP—bupropion, ESC—escitalopram, FLUV—fluvoxamine, PAR—paroxetine, SER—sertraline, TRA—trazodone.

The results of the ATP content and kinetics measurements are shown in Figure 6A,B and Figure 7A,B.

### 3.4. MAO Activity

**Figure 8:**
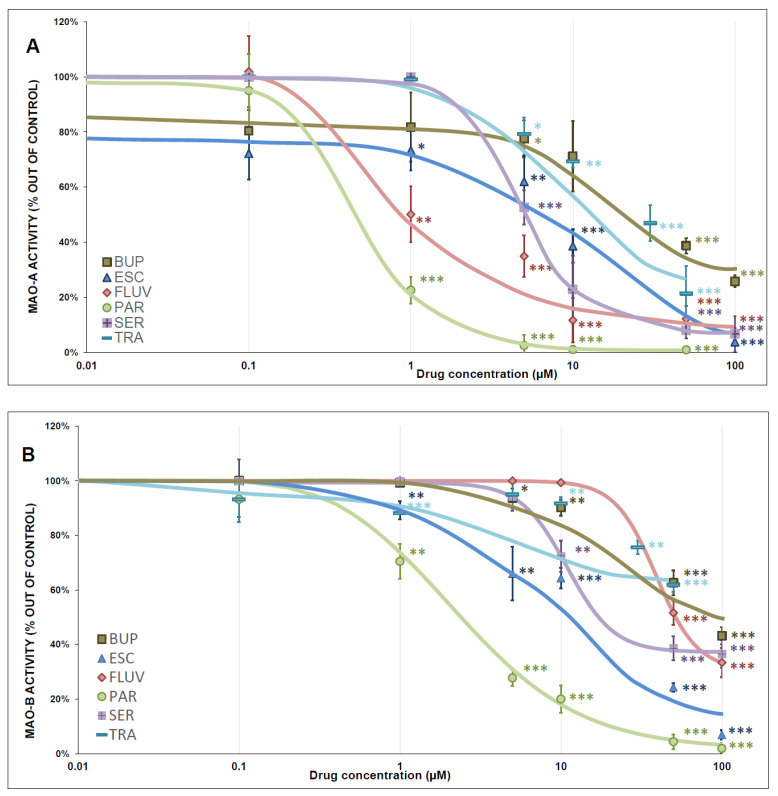
Antidepressant-induced inhibition of MAO activity (MAO-A, MAO-B, (**A**,**B**), respectively). Relative activity is expressed as the percentage difference from the activity of the control sample. A one-sample *t*-test was performed to evaluate statistical significance with the mean control value set at 100% and is expressed as * *p* ˂ 0.05. ** *p* ˂ 0.01. *** *p* ˂ 0.001. Table 2 shows the calculated half-maximal inhibitory concentration (IC_50_), Hill slope, and the residual activity. Drug concentrations are expressed in μmol/L. BUP—bupropion, ESC—escitalopram, FLUV—fluvoxamine, PAR—paroxetine, SER—sertraline, TRA—trazodone.

All tested antidepressants significantly inhibited MAO-A activity: ESC, FLUV, and PAR acted as full inhibitors, and BUP, SER, and TRA acted as partial inhibitors. MAO-B activity was also significantly inhibited by all tested antidepressants: ESC and PAR acted as full inhibitors, and BUP, FLUV, SER, and TRA acted as partial MAO-B inhibitors. The antidepressant-induced MAO-A and MAO-B inhibition curves are shown in Figure 8A,B, and the kinetic parameters (IC_50_, Hill slope, and residual activity) are summarized in Table 2.

### 3.5. Correlations

Several statistically significant correlations were found between measured mitochondrial parameters (complex I, II + III, and IV activities; complex I-linked respiration; complex II-linked respiration; MAO-A and MAO-B activities; complex I-linked ATP content and kinetics; and complex II-linked ATP content and kinetics) using Pearson correlation coefficients. Strong and statistically significant correlations were found between the activities of individual complexes I, II + III, and IV, between the activities of complexes I, II + III, and IV and complex I-linked respiration, between complex II + III and complex II-linked respiration, between complex II-linked ATP kinetics and complex II-linked ATP content, between complex II-linked ATP kinetics and complex II-linked respiration, and between MAO and complex I activities or complex I-linked respiration. Table 3 summarizes the results of the correlation analysis.

## 4. Discussion

We evaluated the antidepressant-induced changes in mitochondrial energy metabolism and MAO-A and MAO-B activities. Any potential mitochondrial impairment (disease- or drug-related) could have an impact on neurotransmission, neuroplasticity, and neurodevelopment. Inefficient energy supply together with increased MAO-A and MAO-B activities could participate in the pathophysiology of psychiatric and neurodegenerative disorders and influence the adverse effects of drugs [23,47,48]. In vitro studies of the mitochondrial effects of antidepressants are essential for improving our knowledge of the pathophysiology of depression, elucidating the mechanisms of the therapeutic and adverse effects of antidepressants, and discovering potential mitochondrial targets for new drugs.

### 4.1. Mitochondrial Enzyme Activity and Respiration

All tested antidepressants (except BUP in complex II + III) significantly inhibited the activities of all ETC complexes. All tested SSRIs were more potent inhibitors of complex I (inhibiting complex I activity by more than 50%) than BUP and TRA. The inhibitory effect of SSRIs was less distinctive in complex II+III activity, with significant inhibition only by PAR (64.3 ± 4.4% at 100 μmol/L, *p* < 0.001) and SER (50.7 ± 1.2% at 100 μmol/L, *p* < 0.001). Overall, the most vulnerable complex was complex IV, and all tested antidepressants decreased its activity below 80% of the control. These findings are in accordance with our previous studies with pharmacologically different antidepressants that potently inhibited mitochondrial respiration and the activities of ETC complexes, especially at high concentrations [34,49].

Complex I-linked respiration was significantly inhibited by all tested antidepressants except BUP, which is in accordance with the inhibition of complex I. Complex I is the main entry and first control point to oxidative phosphorylation (OXPHOS) and is greatly vulnerable to lipophilic molecules and oxidative stress [50]. It is also a rate-limiting unit for oxygen consumption. Inhibition of complex I activity could affect mitochondrial respiration, cause ineffective OXPHOS, and impair ATP production [51]. Inhibition of mitochondrial complexes I and III can be linked to higher ROS production and oxidative damage; moreover, it causes a shift in the production of ATP from mitochondria toward glycolysis, causing increased lactate production [52].

BUP was the only tested antidepressant that partially inhibited complex II-linked respiration and mildly inhibited complex I and IV activities. Our group previously reported that BUP acted as a partial inhibitor of complex I-linked mitochondrial respiration, but the final tested drug concentration reached 1 mmol/L and had no significant effect on the activity of ETC complexes, CS activity, or complex II-linked respiration [49]. There have been very few studies observing the effect of bupropion on cell energy metabolism using cell lines instead of isolated mitochondria, which makes these studies difficult to compare with our results [52,53].

SER and PAR were the strongest inhibitors of complex I, complex II + III, and complex I-linked respiration. They were also very potent inhibitors of complex IV but had no effect on complex II-linked respiration. These findings are partially in accordance with a study observing isolated rat liver mitochondria, where SER (25–100 µmol/L) inhibited the activity of complexes I and V and acted as an OXPHOS uncoupler. The origin of mitochondria as well as applied assays can explain these differences [54]. SER also showed a significant and dose-dependent decrease in oxygen consumption in mitochondria isolated from rat liver, in accordance with our results [55]. Similar to our study, PAR displayed inhibitory properties toward all ETC complexes (nonsignificantly for complex IV) in mitochondria isolated from bovine heart [56].

ESC and FLUV were also very potent complex I activity inhibitors, inhibiting complex IV (FLUV only weakly) and complex I-linked respiration, though with no effect on complex II-linked respiration. ESC showed a similar inhibitory effect in rat brain mitochondria isolated after chronic ESC administration (10 mg/kg for 14 days). The activities of complexes I and II + III were inhibited, while no effect was found on the activity of complex IV, CS, or MDH [50]. The different results for complex IV activity could be explained by chronic ESC administration and the evaluation of different animal tissue.

Study results for FLUV are in accordance with our findings: FLUV caused strong complex I inhibition and mild complex IV inhibition and acted as a partial inhibitor of complex I-linked respiration; it was found to alter the activity of CS, dose dependently affect complex I, and inhibit complex II+II, complex IV, and MDH activities in different rat brain regions [57].

TRA was the most potent inhibitor of complex IV and also inhibited complex I and II+III activities and complex I-linked respiration. Complex IV initiates the final step in the ETC, and its activity has been previously linked with neuronal activation [58]. In another study, TRA acted as an uncoupler of OXPHOS and inhibited oxygen consumption in rat brain homogenate and slices [59].

### 4.2. ATP Production

ATP production and kinetics were determined separately for complex I and complex II to better understand the molecular mechanisms of drug action. Mild but significant decreases in ATP content and kinetics were observed with ESC, FLUV, PAR, and TRA treatments (all at 50 µmol/L); SER treatment resulted in mild increases in ATP content and kinetics.

It was reported previously that different antidepressants decreased the production of mitochondrial ATP: TRA (200 µmol/L) and BUP (0.2–1 mmol/L) significantly decreased ATP contents in HepG2 cells [52,60]; PAR and SER caused collapses in mitochondrial membrane potential and depletion of ATP in H9c2 cells [61,62]; and SER (25–100 µmol/L) was reported to decrease total ATP contents in rat primary hepatocytes, human placental BeWo cells, and human platelets [54,63,64]. Conversely, PAR had no effect on ATP content in murine and human endothelial cells [65]. There are no available studies observing the effect of the chosen antidepressants on isolated mitochondria.

In agreement with the inhibition of complex I activity, all tested antidepressants also inhibited complex I-linked respiration. In contrast, antidepressant-induced changes in complex I-linked ATP content or ATP kinetics were much smaller and were both inhibitory and stimulatory. These findings indicate that ATP production is less sensitive to the in vitro effects of antidepressants than complex I activity and the complex I-linked rate of oxygen consumption in isolated mitochondria. These results support earlier observations that mitochondrial oxygen consumption alone does not appear to be a measure of actual ATP production and that environmental factors can induce variation in mitochondrial efficacy [66]. The cause of variability in the amount of ATP generated per unit of oxygen consumed is not well understood. In the case of proton pumping using complex I, slippage of the proton pumps and the activity of uncoupling proteins can be applied, leading to a change in the coupling efficiency and the attenuation of mitochondrial ROS production [67].

Our results show that, from the point of view of testing the mitochondrial effects of drugs, the measurement of mitochondrial respiratory rates appears to be a more appropriate/sensitive parameter than the measurement of ATP production. Although some antidepressants significantly inhibit the activity of ETC complexes at therapeutically achievable concentrations in the brain, their effects on cellular bioenergetics may be relatively small. It can be speculated that mitochondria are able to use the capacity reserve in the activity of ETC complexes and/or compensatory mechanisms are applied in the OXPHOS system. Knowledge of these mechanisms is necessary to evaluate changes in mitochondrial respiration as markers of drug-induced mitochondrial dysfunction leading to the adverse or therapeutic effects of antidepressants.

### 4.3. MAO Inhibition

All tested antidepressants significantly inhibited both MAO-A and MAO-B activities. Significant levels of inhibition of MAO-A at therapeutically achievable brain concentrations of antidepressants were observed for PAR, FLUV, and ESC, indicating the possibility of enhancing their antidepressant effects through inhibition of the metabolism of monoamine neurotransmitters such as serotonin. Inhibition of MAO-B may enhance the therapeutic effects of PAR and ESC through the reduction in hydrogen peroxide production and perhaps neuroprotective effects unrelated to their primary mechanisms of action [68]. Our results indicate that MAO inhibition could participate in both the antidepressant and neuroprotective effects of the tested antidepressants, which are not classified as MAOIs.

There are no available data describing the effects of the chosen antidepressants on brain-isolated mitochondria. There are studies observing antidepressant-induced MAO-A and MAO-B activity inhibition in rat brain tissue and in female patients with MDD; however, these results are difficult to compare with ours because of the completely different methods employed [69,70,71,72,73,74,75]. Our group previously reported that fluoxetine and citalopram, other SSRI antidepressants, functioned as noncompetitive MAO-A inhibitors and mixed and uncompetitive MAO-B inhibitors. This is in accordance with our current results, considering the structural similarities among the SSRI groups (except FLUV), with the result that MAO-inhibitory properties are expected among these drugs [76].

### 4.4. Correlations

Correlations between the measured mitochondrial parameters reflect the interconnection and balance of the individual components of the OXPHOS system. The significant positive correlations between the activities of complexes I, II + III, and IV confirmed that the activity of a certain ETC complex reflects drug-induced changes in the activities of other complexes and/or that the activities of individual complexes are modified by the antidepressant in the same manner.

The significant positive correlations between the activities of individual ETC complexes and complex I-linked respiration and no or negative correlations between the activities of individual ETC complexes and complex II-linked respiration confirmed that complex I-linked respiration is primarily regulated by the activities of respiratory complexes, while complex II-linked respiration appears to be more regulated by other components of the OXPHOS system (most likely influencing the availability of the electron donor FADH_2_). This approach is also supported by the observation of a significant negative correlation between complex II-linked ATP kinetics and complex II-linked respiration, reflecting the finding that the observed antidepressant-induced reduction in complex II-linked ATP kinetics is not accompanied by reduced complex II-linked respiration. The negative correlation between complex II-linked respiration and complex II-linked ATP kinetics is most likely correlation without causation, because: (i) the only antidepressant that slightly inhibited complex II-linked respiration was BUP, which had no Inhibitory effect towards complex II-linked ATP kinetics; and (ii) the inhibitory effects of the other antidepressants tested were not associated with changes in complex II-linked respiration.

The lack of correlation between complex I-linked respiration and complex I-linked ATP content supports the existence of variability between oxygen consumption and ATP production, as discussed above (Section 4.2). The significant correlations between MAO activity and complex I activity and between MAO activity and complex I-linked respiration, but not between MAO activity and complex II+III or complex IV activity, indicate the possible existence of an association between MAO activity and complex I activity. It can be assumed that similar changes in the activities of MAO and complex I are a consequence of drug-induced conformational changes of the enzyme and/or affecting protein–protein interactions between MAO units or between subunits of complex I.

### 4.5. Study Limitations

In this study, isolated pig brain mitochondria were used as previously described; this in vitro model is suitable for investigating mitochondrial drug effects and allows more accurate and closer recognition of the mitochondrial mechanisms of action of antidepressants [35]. However, the model cannot cover regulatory and compensatory brain mechanisms that could be observed in in vivo measurements.

Significant changes in the measured mitochondrial parameters were often observed even with high concentrations of antidepressants. Therefore, measurements of the effects of drugs on mitochondrial parameters had to be performed over wide concentration ranges, including therapeutic plasma/brain concentrations of antidepressants. The recommended target plasma levels are 3.50–6.30 µM for BUP, 0.05–0.23 µM for ESC, 0.47–0.94 µM for FLUV, 0.21–0.36 µM for PAR, 0.03–0.16 µM for SER, and 1.75–4.03 µM for TRA [77,78]. In our study, the final concentration range of antidepressants used in the experiments was 0.125–100 µmol/L. Most antidepressants are molecules that are amphiphilic and cationic, and they tend to accumulate in subcellular organelles, membranes, and the brain. Moreover, the mitochondrial matrix facilitates the accumulation of xenobiotics inside mitochondria. The rates of mitochondrial replacement might vary from days to weeks, providing enough time for xenobiotic accumulation to cause drug-induced mitochondrial changes [60,79,80]. Almost all tested antidepressants (no PAR data were found) showed brain accumulations in animal and human studies of brain-to-plasma ratios [81,82,83,84,85,86]. Significant changes in mitochondrial function observed at high concentrations of the tested antidepressants should be interpreted with caution, recognizing that the results cannot be directly extrapolated to clinical situations. However, the findings from these in vitro studies provide important preliminary information for further in vivo research using appropriate animal models and clinical trials, which are necessary for clinical validation and understanding of the relevance of the observed in vitro effects of high doses of antidepressants.

The rationale for studying the in vitro mitochondrial effects of tested antidepressants in a wide range of concentrations, significantly exceeding the therapeutic plasma concentrations of these drugs, includes: (i) safety and toxicity assessment (recognition of mitochondrial adverse effects that may occur after overdose or local accumulation of the drug); (ii) elucidation of the mechanisms by which the tested drugs exert their effects on cellular function with a focus on mitochondrial function; (iii) comparative analysis of mitochondrial changes at low and high concentrations of antidepressants in order to recognize and understand dose-dependent effects and to determine potential toxic doses; and (iv) theoretical contributions in the field of revealing the mitochondrial response to extremely high concentrations of antidepressants.

A limitation of this study is the lack of information on the effects of antidepressants on mitochondrial morphology and oxidative stress. Antidepressant-induced changes that may be related to changes in mitochondrial morphology have been described [54,87,88,89]. This suggests a potential effect of antidepressants on mitochondrial morphology, which is associated with mitochondrial dysfunction and increased oxidative stress. The effect of antidepressants on oxidative stress, measured as increased production of ROS, lipid peroxidation, or decreased activity of antioxidant enzymes has been described [90,91,92]. Therefore, antidepressants have the potential to affect mitochondrial morphology and regulate the oxidative stress, and these effects should be further investigated for a full understanding of their therapeutic effects or side effects.

### 4.6. Possible Clinical Impact

The most frequent adverse effect of SSRIs is nausea, and the overall incidence was similar for all SSRIs. PAR probably has the worse tolerability profile of all SSRIs [93,94]. Considering that SER and PAR were the most potent inhibitors of mitochondrial function and, according to the literature, also the drugs with the lowest tolerability profiles among the tested SSRIs, this mitochondrial inhibition could be connected to their adverse effects, especially through complex II+III activity inhibition [30,93].

BUP has similar efficacy but a better tolerability profile than SSRIs, usually causes lower rates of sexual dysfunction, and it is more likely to cause weight loss [95,96]. TRA is generally considered to be as effective as SSRIs and less likely to cause sexual dysfunction or insomnia [97].

Mixed results have been found for the effect of antidepressants on cognition, probably through complex mechanisms involving monoamine transporters, receptors, and degradative enzymes (including MAO). A meta-analysis showed that antidepressant treatment had a modest beneficial effect on cognitive function in depressed patients, with the largest effect mediated by SSRIs. This was not shown for non-depressed participants [98]. It was published that long-term SSRI treatment of depressed patients with mild cognitive impairment significantly delayed progression to Alzheimer’s dementia compared with short-term SSRI treatment, other antidepressants, or no treatment, and compared with patients with mild cognitive impairment without a history of depression [99]. A systematic review confirmed the procognitive effect of newer SSRIs, with the best results for ESC. Cognitive improvement was also confirmed for BUP, whose procognitive effect is mainly attributed to its noradrenergic mechanism of action [100]. TRA is useful in the control of agitation and insomnia in AD. A systematic review analyzing the effect of TRA on cognition found that acute TRA treatment may be associated with cognitive impairment, but long-term treatment tends to prevent cognitive decline. It is important to note, that this effect is related to the effect of TRA on the unfolded protein response pathway [101]. A cohort study found no cognitive benefit in patients with dementia taking TRA compared to other antidepressants [102].

## 5. Conclusions

The results of this in vitro study showed that SSRIs, BUP, and TRA significantly altered OXPHOS system functioning. All tested antidepressants (except BUP in complex II + III) significantly decreased ETC complexes activities at all tested concentrations. The most potent inhibitors of complex I and II + III activities were PAR and SER, and the strongest complex IV inhibitor was TRA. All tested antidepressants except BUP significantly reduced complex I-linked respiration (PAR and FLUV at very high concentrations). The SSRIs were the strongest inhibitors of complex I and II+III activities and complex I-linked respiration compared to BUP and TRA. BUP was the only tested antidepressant with some inhibitory activity against complex II-linked respiration. Based on our results, SSRIs affect mitochondrial ETC complexes and respiration differently than BUP and TRA. Considering that all tested antidepressants showed inhibitory properties against OXPHOS, they can participate in drug-induced mitochondrial dysfunction, which can endanger neuronal adaptation and body homeostasis, especially at high doses. Antidepressants did not significantly affect total ATP content or kinetics, suggesting that ATP production is not directly dependent only on ETC enzymes activities; engagement of mitochondrial capacity reserve or potential OXPHOS compensatory mechanisms may occur.

Understanding the subcellular mechanisms of action of antidepressants and evaluating their effects on mitochondrial parameters is a very important step in elucidating how drug-related mitochondrial changes contribute to their therapeutic and/or adverse effects. Mitochondrial dysfunction could be a treatment response modulator and/or a predictor of patient responsiveness, which might help pharmacoresistant patients in choosing an effective pharmacotherapy. Based on our study results, it is evident that these drugs have MAO-inhibiting properties that could participate in their antidepressant, procognitive, and neuroprotective effects. These mechanisms should be further investigated, especially in in vivo studies.

## Figures and Tables

**Table 1:** Inhibitory parameters of antidepressant-induced changes in complex I- and complex II-linked respiration in isolated mitochondria.

Complex I-Linked Respiration				
Drug	IC_50_ (µmol/L)	Hill Slope	Residual Activity (rel.u.)	Inhibition
escitalopram	0.14 ± 0.08	1.39 ± 1.05	0.774 ± 0.015	partial
fluvoxamine	15.84 ± 2.57	3.09 ± 1.51	0.817 ± 0.017	partial
paroxetine	26.71 ± 6.73	1.27 ± 0.36	0.672 ± 0.029	partial
sertraline	12.38 ± 0.75	2.05 ± 0.23	0.179 ± 0.031	partial
trazodone	0.45 ± 0.19	1.10 ± 0.43	0.815 ± 0.015	partial
complex II-linked respiration				
bupropion	10.58 ± 4.49	1.17 ± 0.44	0.690 ± 0.045	partial

The mean ± SEM is used to express the values obtained from four independent measurements. IC_50_ refers to the half-maximal inhibitory concentration.

**Table 2:** The inhibition of monoamine oxidase activity (MAO-A and MAO-B) induced by antidepressants.

MAO-A				
Drug	IC_50_ (µmol/L)	Hill Slope	Residual Activity (rel.u.)	Inhibition
bupropion	20.15 ± 5.68	1.36 ± 0.33	0.235 ± 0.050	partial
escitalopram	8.78 ± 2.27	0.99 ± 0.29	0.005 ± 0.057	full
fluvoxamine	0.99 ± 0.23	0.91 ± 0.17	0.079 ± 0.041	full
paroxetine	0.47 ± 0.06	1.65 ± 0.22	0.007 ± 0.017	full
sertraline	4.91 ± 0.23	2.37 ± 0.39	0.074 ± 0.017	partial
trazodone	13.16 ± 2.55	1.23 ± 0.22	0.184 ± 0.042	partial
**MAO-B**				
bupropion	31.54 ± 3.32	1.68 ± 0.20	0.410 ± 0.018	partial
escitalopram	15.16 ± 2.23	1.13 ± 0.14	0.038 ± 0.032	full
fluvoxamine	39.04 ± 2.63	3.39 ± 1.35	0.306 ± 0.034	partial
paroxetine	2.19 ± 0.21	1.06 ± 0.10	0.015 ± 0.021	full
sertraline	10.95 ± 0.59	3.00 ± 0.68	0.372 ± 0.014	partial
trazodone	29.73 ± 5.77	2.75 ± 1.35	0.560 ± 0.079	partial

The mean ± SEM is used to express the values obtained from four independent measurements. IC_50_ refers to the half-maximal inhibitory concentration.

**Table 3:** Correlation coefficients for antidepressant-induced changes in mitochondrial parameters.

		Complex I Activity	Complex II + III Activity	Complex IV Activity	Complex I-Linked ATP Content	Complex I-Linked ATP Kinetics	Complex II-Linked ATP Content	Complex II-Linked ATP Kinetics	Complex I-Linked Respiration	Complex II-Linked Respiration
complex II + III activity	*r*	*** 0.58	-	-	-	-	-	-	-	-
	*N*	153								
complex IV activity	*r*	*** 0.75	*** 0.66	-	-	-	-	-	-	-
	*N*	64	64							
complex I-linked ATP content	*r*	−0.16	0.03	* −0.32						
	*N*	53	53	53	-	-	-	-	-	-
complex I-linked ATP kinetics	*r*	* −0.35	0.14	−0.22	−0.08					
	*N*	46	46	46	44	-	-	-	-	-
complex II-linked ATP content	*r*	0.12	0.19	0.00	0.06	0.11				
	*N*	56	56	56	52	46	-	-	-	-
complex II-linked ATP kinetics	*r*	0.04	** 0.42	−0.03	0.12	0.19	*** 0.50			
	*N*	45	45	45	45	43	45	-	-	-
complex I-linked respiration	*r*	*** 0.51	*** 0.57	*** 0.66	−0.16	−0.16	−0.01	0.10		
	*N*	82	82	55	52	43	52	45	-	-
complex II-linked respiration	*r*	−0.05	*** −0.61	* −0.43	0.03	−0.21	−0.26	*** −0.75	−0.09	
	*N*	54	54	27	24	21	25	21	294	-
MAO-A activity	*r*	*** 0.48	0.02	0.06	0.04	0.12	0.26	0.28	* 0.23	0.12
	*N*	71	71	52	50	42	51	44	100	74
MAO-B activity	*r*	*** 0.58	−0.04	* 0.30	−0.12	−0.20	−0.06	0.06	*** 0.37	0.15
	*N*	87	87	63	53	46	56	45	104	76

The Pearson correlation coefficient (*r*) was used to assess the linear correlation between mitochondrial parameters measured at different concentrations of antidepressants. Statistical significance is indicated as * *p* ˂ 0.05. ** *p* ˂ 0.01. *** *p* ˂ 0.001. The number of measurements is indicated as *N*.

## Data Availability

All of the data is contained within the article and the supplementary materials.

## Supplementary Materials

The following supporting information can be downloaded at: https://www.mdpi.com/article/10.3390/antiox12061208/s1. Table S1. The changes in citrate synthase (CS) and malate dehydrogenase (MDH) activities induced by antidepressants.

## Abbreviations

BUP	bupropion
CS	citrate synthase
ESC	escitalopram
ETC	electron transport chain
FLUV	fluvoxamine
IC_50_	half-maximal inhibitory concentration
MAOI	monoamine oxidase inhibitor
MAO	monoamine oxidase
MDD	major depressive disorder
MDH	malate dehydrogenase
NADH	Reduced nicotinamide adenine dinucleotide
OXPHOS	oxidative phosphorylation
PAR	paroxetine
ROS	reactive oxygen species
SD	standard deviation
SEM	standard error of the mean
SER	sertraline
SSRI	selective serotonin reuptake inhibitor
TRA	trazodone
