# Supplementary material for: Different Effects of SSRIs, Bupropion, and Trazodone on Mitochondrial Functions and Monoamine Oxidase Isoform Activity
Source: Antioxidants (Basel). 2023 Jun 2;12(6):1208. doi: 10.3390/antiox12061208 (PMC10294837; doi:10.3390/antiox12061208)
Supplement: Supplementary file 1 [file antioxidants-12-01208-s001.zip › antioxidants-2358620-supplementary.pdf]

Different Effects of SSRIs, Bupropion, and Trazodone on Mitochondrial Functions and Monoamine Oxidase Isoform Activity

Matej Ľupták [1,*], Zdeněk Fišar [2] and Jana Hroudová [1,2,*]

[1] Institute of Pharmacology, First Faculty of Medicine, Charles University and General University Hospital in Prague, Albertov 4, 128 00 Prague, Czech Republic
[2] Department of Psychiatry, First Faculty of Medicine, Charles University and General University Hospital in Prague, Ke Karlovu 11, 120 00 Prague, Czech Republic; zfisar@lf1.cuni.cz
* Correspondence: mat.luptak@gmail.com (M.L'.); jana.hroudova@vfn.cz (J.H.); Tel.: +420-224965122 (M.L'. & J.H.)

Content:

- The effect of antidepressants on citrate synthase (CS) and malate dehydrogenase (MDH) activities (Table S1)

Table S1: The effect of antidepressants on citrate synthase (CS) and malate dehydrogenase (MDH) activities

drug	concentration	CS (% of control)	N	MDH (% of control)	N
bupropion	10 µM	*105.6 ± 1.29	3	99.2 ± 2.90	3
bupropion	100 µM	99.5 ± 0.93	3	99.6 ± 2.72	3
escitalopram	10 µM	102.4 ± 3.60	3	98.0 ± 6.10	3
escitalopram	100 µM	*97.2 ± 1.52	3	99.6 ± 2.64	3
fluvoxamine	10 µM	*106.3 ± 1.22	3	99.9 ± 0.77	3
fluvoxamine	100 µM	100.5 ± 1.23	3	98.3 ± 2.86	3
paroxetine	10 µM	*105.8 ± 1.69	3	100.1 ± 2.22	3
paroxetine	100 µM	*98.4 ± 0.85	3	97.9 ± 1.71	3
sertraline	10 µM	*105.2 ± 1.99	3	99.2 ± 2.90	3
sertraline	100 µM	97.9 ± 1.83	3	97.8 ± 6.35	3
trazodone	10 µM	109.0 ± 6.02	3	99.2 ± 2.90	3
trazodone	100 µM	**96.0 ± 0.80	3	99.0 ± 1.62	3

The values are expressed as the mean ± SD for 3 independent measurements. Statistical significance was tested using a one sample *t*-test that mean value is equal to 100% and is expressed as *$p < 0.05$. **$p < 0.01$. N is number of measurements.
